# Tempo-Spatial Variations of Ambient Ozone-Mortality Associations in the USA: Results from the NMMAPS Data

**DOI:** 10.3390/ijerph13090851

**Published:** 2016-08-26

**Authors:** Tao Liu, Weilin Zeng, Hualiang Lin, Shannon Rutherford, Jianpeng Xiao, Xing Li, Zhihao Li, Zhengmin Qian, Baixiang Feng, Wenjun Ma

**Affiliations:** 1 Guangdong Provincial Institute of Public Health, Guangdong Provincial Center for Disease Control and Prevention, No. 160, Qunxian Road, Panyu District, Guangzhou 511430, China; gztt_2002@163.com (T.L.); letitiazeng@foxmail.com (W.Z.); linhualiang2002@163.com (H.L.); jpengx@163.com (J.X.); lixing.echo@foxmail.com (X.L.); zhihaoli1990@163.com (Z.L.); fengbaixiang@126.com (B.F.); 2 Environment and Health, Guangdong Provincial Key Medical Discipline of Twelfth Five-Year Plan, Guangzhou 511430, China; 3 Centre for Environment and Population Health, Griffith University, Brisbane 4111, Australia; s.rutherford@griffith.edu.au; 4 Department of Epidemiology, College for Public Health and Social Justice, Saint Louis University, St. Louis, MO 63104, USA; zqian2@slu.edu

**Keywords:** air pollution, ambient ozone, mortality, time-series study, seasonal variation, USA

## Abstract

Although the health effects of ambient ozone have been widely assessed, their tempo-spatial variations remain unclear. We selected 20 communities (ten each from southern and northern USA) based on the US National Morbidity, Mortality, and Air Pollution Study (NMMAPS) dataset. A generalized linear model (GLM) was used to estimate the season-specific association between each 10 ppb (lag0-2 day average) increment in daily 8 h maximum ozone concentration and mortality in every community. The results showed that in the southern communities, a 10 ppb increment in ozone was linked to an increment of mortality of −0.07%, −0.17%, 0.40% and 0.27% in spring, summer, autumn and winter, respectively. For the northern communities, the excess risks (ERs) were 0.74%, 1.21%, 0.52% and −0.65% in the spring, summer, autumn and winter seasons, respectively. City-specific ozone-related mortality effects were positively related with latitude, but negatively related with seasonal average temperature in the spring, summer and autumn seasons. However, a reverse relationship was found in the winter. We concluded that there were different seasonal patterns of ozone effects on mortality between southern and northern US communities. Latitude and seasonal average temperature were identified as modifiers of the ambient ozone-related mortality risks.

## 1. Introduction

Ozone is a key component in the troposphere and plays an important role in air quality, atmospheric oxidizing capacity, and climate change [1,2]. Surface ozone, especially in urban areas, mainly comes from photochemical reactions between oxides of nitrogen (NO_x_) and volatile organic compounds (VOCs) in the presence of sunlight. In the past decades, associated with rapid urbanization and industrialization processes, increased anthropogenic emissions of NO_x_ and VOCs has led to higher surface ozone concentration in some regions of the world [1,3,4,5,6]. The Intergovernmental Panel on Climate Change (IPCC) fifth assessment report in 2013 projected with high confidence that under the Representative Concentration Pathways (RCP)8.5 scenario the average global background surface ozone concentration would rise by about 8 ppb (25% of current levels) by the year 2100 relative to RCP4.5 or RCP6.0 scenarios [7]. These data imply that ambient ozone pollution will continue to be a global environmental problem throughout the 21st century.

Exposure to ozone has been definitively linked to a host of adverse health effects, including increased hospital admission rates and emergency department visits, exacerbation of chronic diseases and mortality [8,9,10,11]. Furthermore, a seasonal pattern has been observed in the association between ambient ozone and human health. For example, studies conducted in the USA and Europe found higher effects of ozone in the warm season than in the cold season [11,12,13,14,15]. However, other studies, especially from southern China, revealed more pronounced associations between ozone and mortality in the cold season than in the warm season [16,17,18,19,20,21,22,23]. One of our recent studies also identified that more pronounced effects of ozone on mortality were found in the cold season of Guangzhou, China [24]. Results across different studies indicate that the seasonal patterns of ozone effects on mortality differ between regions, indicating geographic variations of ozone effects [13,25]. For instance, Ren et al. observed more pronounced effects of ambient ozone on mortality in the northeast than in the southeast region of the USA [11]. These previous findings show that the health impacts of ozone might have both seasonal and geographic heterogeneities. However, these findings were observed in independent studies of seasonal and geographical influence, and no studies have been conducted to simultaneously assess the seasonal and geographic variations of ozone effects on health. This type of study will help to provide a more comprehensive understanding of the ambient ozone effects on human health, better understand potential risks in the future as a result of warming temperatures and provide useful evidence for designing ozone pollution control strategies.

The US National Morbidity, Mortality, and Air Pollution Study (NMMAPS) data covers 108 urbanized communities across the USA, containing time series data for health outcomes, air pollution and weather conditions between 1 January 1987 and 31 December 2000 [26]. These communities vary geographically and have different climatic types which may influence people’s exposure pattern, a particularly important issue considering ozone is an outdoor pollutant that is generated in the atmosphere in the presence of sunlight. Therefore, utilizing this NMMAPS data provided an opportunity to simultaneously assess the seasonal and geographic variations of ozone effects on health.

For this study, we selected 20 mainland USA communities that had high quality data provided by the NMMAPS. We aimed to separately assess the effects of ambient ozone on non-accidental mortality in every season of both southern and northern communities, and explore the impacts of city-specific characteristics on the ozone effects on mortality.

Adjustment for the potential confounding effects of other air pollutants is an important issue in time-series studies for air pollutants. However, in the communities included in the NMMAPS dataset, only the particulate matter with an aerodynamic diameter less than 10 mm (PM_10_) was measured every six days for most communities, and the proportion of missing data for PM_10_ was high. In addition, some studies have shown that PM_10_ did not confound ozone effect estimates using the NMMAPS data [11,27]. Therefore, we did not adjust for PM_10_ in this study.

## 2. Experimental Section

### 2.1. Study Setting

**Figure 1:**
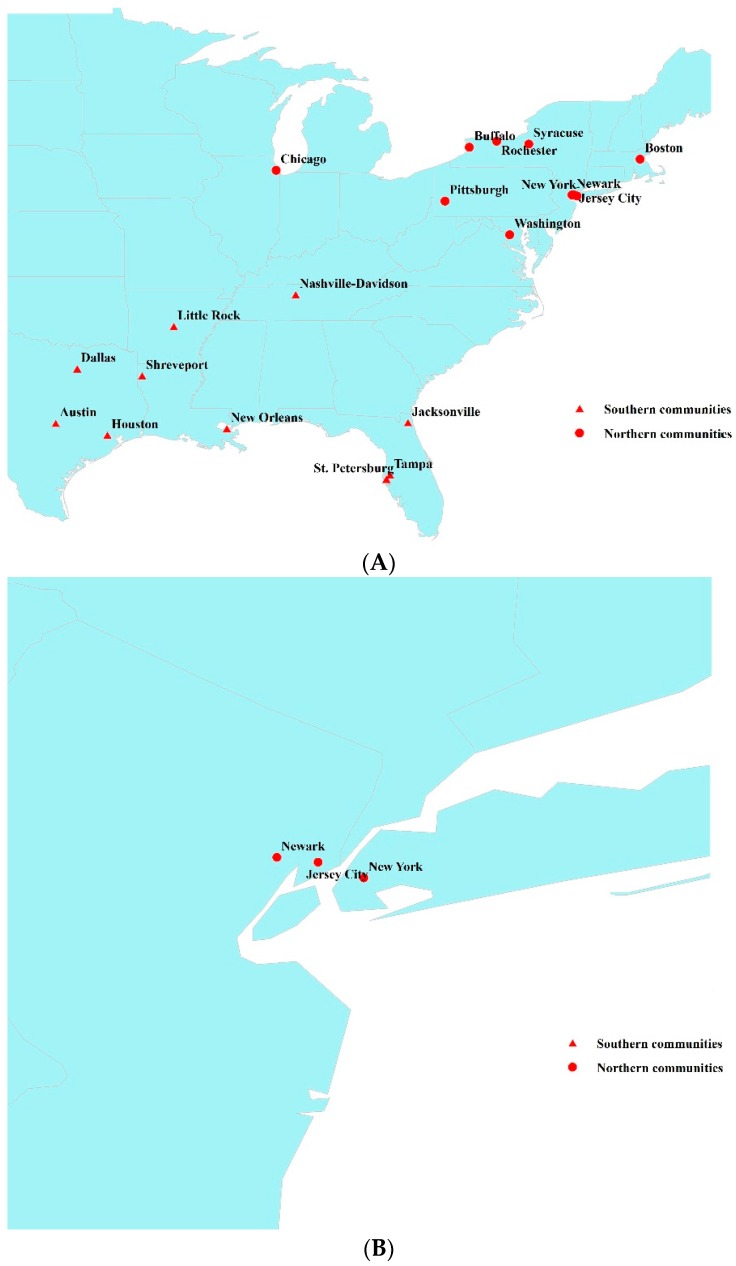
Distribution of 20 study communities in the USA. Note: This figure depicts the distribution of selected communities in the USA through ArcGis (ArcMap 9.3, Environmental Systems Research Institute, Redlands, CA, USA). (**A**): The distribution of all included communities; (**B**): The location of three northern communities that are located closely each other.

This study was based on the NMMAPS dataset for 1987–2000 obtained from the National Center for Health Statistics [28]. The dataset included information on air pollution, mortality and weather conditions for 108 large communities distributed across the USA. In order to achieve the present study aims, we randomly selected ten communities from a region that experienced a humid, subtropical climate (in this case, the southern region), and ten communities from a region with a temperate, continental climate (in this case, the northern region). High quality data were available for analysis in all the chosen communities. Along with direct differences in climate conditions, people in these two regions may have different activity models, which in turn might affect their exposure to ambient air pollutants [21]. The distribution of all selected 20 communities is shown in Figure 1 and their general information is shown in Table 1 and Figure S1. To test the seasonal variations of ozone effects on mortality in different regions, all data were divided into four seasons: spring (March−May), summer (June−August), autumn (September−November) and winter (December−February).

### 2.2. Data Collection

The health outcome for each selected community was daily non-accidental mortality which was categorized using codes below 800 from the International Classification of Diseases Ninth Revision (ICD-9) and codes below “S” from the International Statistical Classification of Diseases 10th Revision (ICD-10). Ninth Revision codes were used for 1987–1998 and the ICD-10 for 1999 and 2000 [27]. We chose total non-accidental mortality as the health outcome since it is the most common health outcome used in previous time-series studies [8,9,10,11,12,13,14,15,16]. In addition, population size in some included communities that were relatively very small and for some, such as Shreveport and Little Rock, the number of daily deaths was very low. Employing total non-accidental mortality data increased statistical power in the statistical analyses.

Air pollution data for the daily 8 h maximum ozone concentration in each community was provided to the NMMAPS database by the USA Environmental Protection Agency (EPA) Aerometric Information Retrieval Service (now called the Air Quality System database). To avoid a disproportional impact of outliers on analytic outcomes, a 10% trimmed mean was used to average across monitors after correction for yearly averages for each monitor [27]. We chose daily 8 h maximum concentration as the ozone concentration indicator because previous studies indicated that it has a stronger association with health outcomes compared with other metrics, and appears to be a more appropriate metric for investigating health effects of ambient ozone exposure [22,29].

Daily meteorological data for each community was provided to the NMMAPS database by the National Climatic Data Center. Measurements from multiple weather stations were averaged to provide weather variables representing each community. Meteorological data included daily mean temperature (TM, °C) and relative humidity (RH, %). Other information was obtained from the NMMAPS database.

### 2.3. Statistical Analysis

ArcGis (ArcMap 9.3, Environmental Systems Research Institute, Redlands, CA, USA) technology was employed to describe the distribution of selected communities in the USA.

A two-stage model was employed to examine the effect of ozone on mortality. In the first stage, we used a generalized linear model (GLM) with a log link and Poisson error to smooth day-to-day fluctuations in each selected community. The model is expressed as:

Log[*E*(*Y_t_*)] = α + βZ + *ns*(TM, df) + *ns*(RH, df) *+ ns*(time, df) + ηDOW (1)

where *t* is the day of observation; *E*(*Y_t_*) is the expected number of deaths on day *t*; Z represents daily 8 h maximum ozone concentration. β is the coefficients for *T.* According to Bell et al.’s finding that the health impacts of ozone were mainly concentrated up to a lag of two days [27], we employed a three-day moving average (lag0-2) concentration of ozone to effectively capture its overall effect that is estimated by an excess mortality risk (ER) for every 10 ppb increment in ozone concentration.

To control for confounding effects of TM we employed a natural cubic spline function (*ns*, *df* = 3) to estimate the non-linear effect of TM [30]. A contemporaneous effect (lag0-2 day average) of TM with ozone was controlled for in the model. Consistent with previous studies, the *df* for TM was set to 3 [24,31]. Similarly, the *ns* (*df* = 3) model was also used to control for the confounding effects of RH. *Time* in *ns* (time, *df*) equals 1, 2, 3, … 5114 (day of the year 1987–2000). We used 7 (*df*) per year for the overall smooth function of time [32]. Day of week (DOW) was used as a factor variable, and η is a vector of coefficients.

In the second stage, a series of meta-analyses were used to estimate the summary effects of the ozone on mortality in each season for southern and northern communities, respectively [33,34]. The Cochran’s Q statistic was calculated to test the possible heterogeneity of ozone effects between communities. If the *p*-value for heterogeneity test was <0.05, a random-effects model would be selected, otherwise a fixed-effects model would be selected to estimate the summary effects [35,36]. Forest plots and pooled ERs were reported. We also employed several meta-regression analyses to explore the impacts of latitude, seasonal average TM and RH on the ozone effects in all included communities. We calculated the mortality effect (RR) change per 1° increment in latitude, 1 °C increment in TM and 1% increase in RH.

A series of sensitivity analyses were performed to test the robustness of our results by changing the *df* of smoothness of time per year and lag days (lag0-1 and lag0-3). We changed the *df* from 5 to 9 per year in the GLM models. In order to test the impacts of ozone concentration on effects on mortality, we estimated the effects of ozone in communities with the maximum concentrations of ozone in each season at both northern and southern regions.

All statistical tests were two-sided, and *p* < 0.05 was considered statistically significant. We used R software (version 2.15.2; R Development Core Team 2012, http://www.R-project.org/) to analyze the data. The “dlnm” package was used to fit Poisson regression [37]. The “Metafor” package was used to fit meta-analysis [33].

## 3. Results

Table 2 presents the distributions of mortality, weather statistics and ozone concentrations in the full year and for every season of the selected southern and northern communities in the USA. For the entire year, the average number of daily non-accidental deaths in the southern and northern communities was 21.0 and 45.5, respectively. The corresponding two regional average TMs were 20.0 °C and 11.1 °C, and the average concentrations of daily 8 h maximum ozone were 39.7 and 32.1 ppb. We observed obvious variations in average mortality, TM, RH and ozone concentration between the southern and northern communities in each season. Detailed information is provided in Table 2.

**Figure 2:**
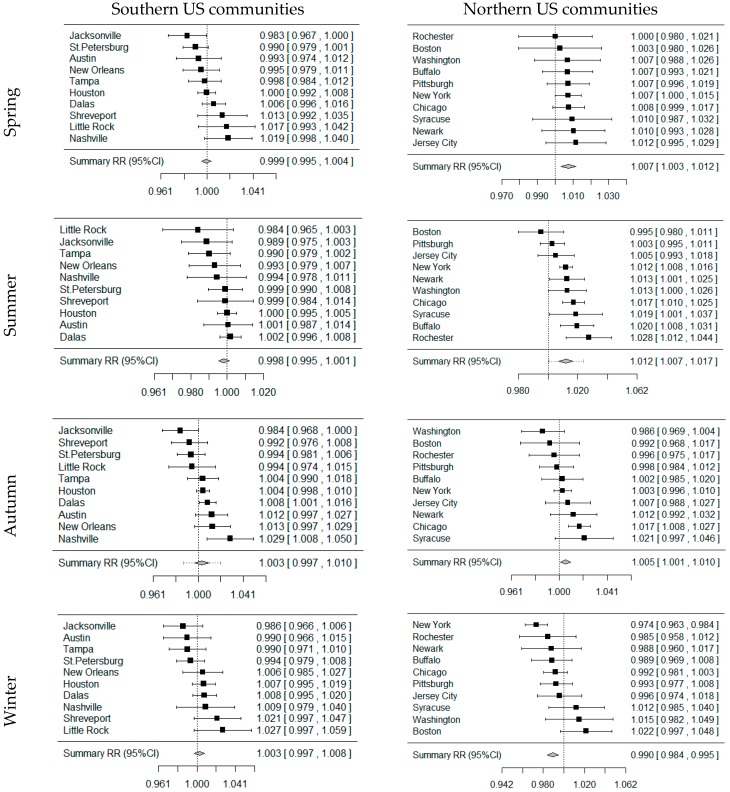
The summary effects of ambient ozone on mortality in each season of southern and northern US communities. *Note*: All results were adjusted for daily mean temperature (TM), time, day of week and relative humidity (RH).

Forest plots in Figure 2 show the summary effects of ambient ozone on mortality in each season of southern and northern communities. In the southern communities, ambient ozone had more pronounced associations with mortality in autumn and winter than in spring and summer. Particularly, in autumn a 10 ppb increase in lag0-2 day average ozone concentration was significantly associated with an increase of 0.40% (95% CI: 0.05% ~ 0.75%) mortality risk. In contrast, we observed a different pattern of ambient ozone-related effects in the northern communities. Stronger effects of ozone were found in spring, summer and autumn than in winter. Ozone exposure in summer had the strongest effect on mortality among the four seasons. A 10 ppb increase in lag0-2 day average ozone concentration in summer was associated with an increase of 1.21% (95% CI: 0.72% ~ 1.71%) mortality risk (Table S1).

**Figure 3:**
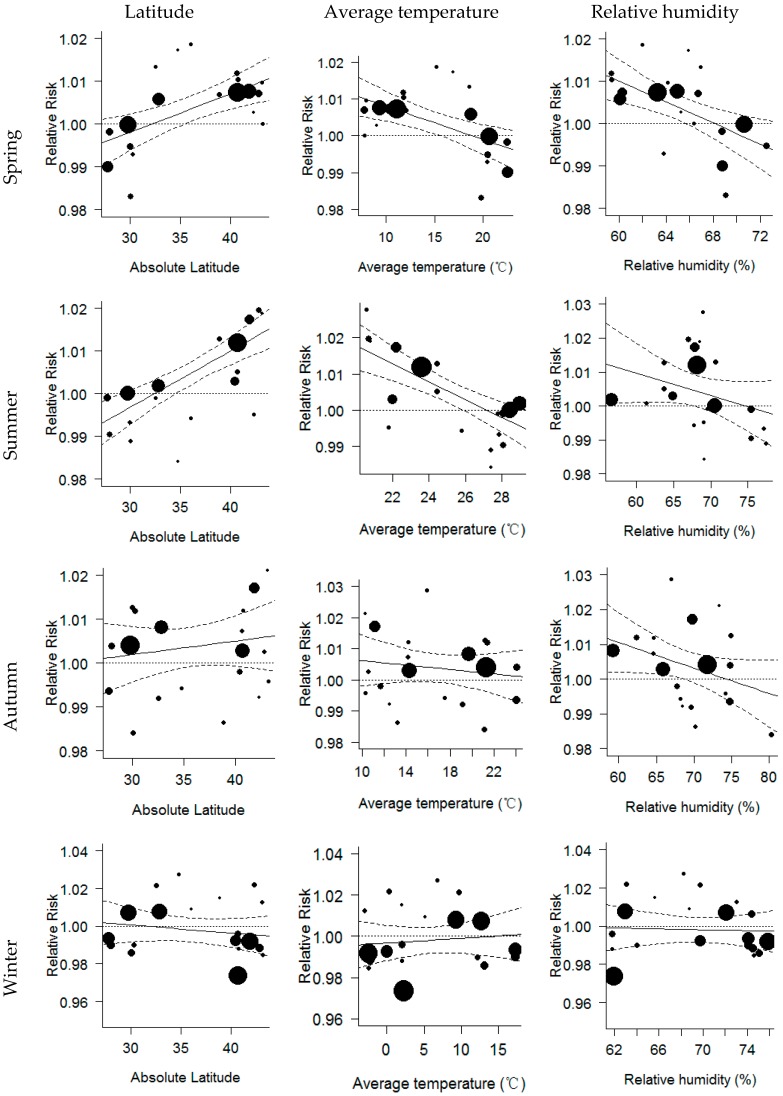
Meta-regression analysis on the associations of seasonal relative risk of ozone on mortality with city-specific absolute latitude, average temperature and RH.

The meta-regression analyses show that city-specific latitude, TM and RH may be important modifiers of ozone effects on mortality. For example, in the spring season, the ozone effect was positively related to latitude increase (β = 0.09, 95% CI: 0.04 ~ 0.14), and negatively related to TM (β = −0.09, 95% CI: −0.15 ~ −0.03) as well as RH (β = −0.11, 95% CI: −0.20 ~ −0.05) in all communities. Similar results were also found in the summer and autumn seasons. However, in the winter season, reverse results were found. The ozone effect was negatively related to latitude increase (β = −0.04, 95% CI: −0.15 ~ 0.07), and was positively related to TM increase (β = 0.02, 95% CI: −0.07 ~ −0.12) in all communities, but these relationships were not statistically significant (Figure 3 and Table S2).

**Figure 4:**
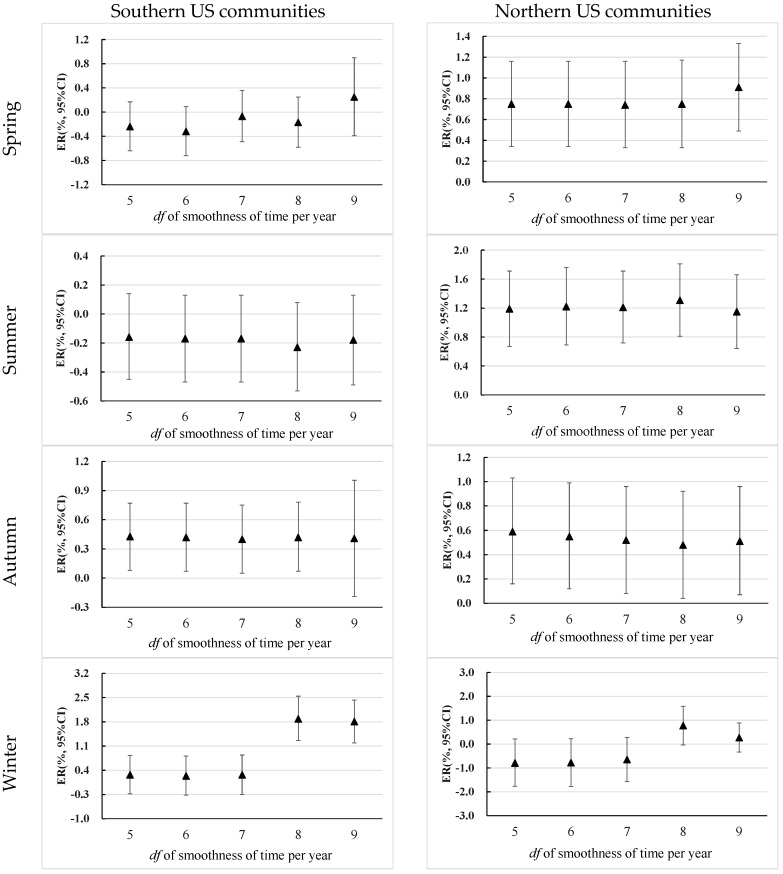
Sensitivity analysis on the effects of ozone on mortality with different *df* of smoothness of time per year. *Note*: All ERs were adjusted for daily TM, time, day of week and RH. National Morbidity Mortality and Air Pollution Study (NMMAPS) database.

Sensitivity analyses indicate that the results were generally robust to changing the *df* of smoothness of time per year, but there is an exception (Figure 4). In the analysis that examined the effect of ozone on mortality in the winter of southern communities, the ER for each 10 ppb increment in ozone concentration significantly increased from 0.27% (95% CI: −0.15% ~ 0.07%) to 1.89% (95% CI: 1.26% ~ 2.54%) when changing the *df* from 7 to 8 per year. However, the ER did not change significantly when changing the *df* of time in the range of 5–7 per year. We did not find significant changes of ozone effects when altering the lag days of ozone exposure (lag0-1, lag0-2 and lag0-3), either. We observed similar seasonal and regional patterns of ozone effects in the communities with the maximum concentrations at both northern and southern regions. For example, a significant effect of ozone on mortality was observed in the autumn season of Dallas (southern community) and in the summer season of Washington (norther community) (Table S3).

## 4. Discussion

Ambient ozone is a compelling environmental problem that has received significant attention all over the world [7]. Assessing the seasonal and geographic variations of ozone effects on human health can provide additional information for making policy on ambient ozone control particularly in a climate-changing environment where projections suggest a significant increase in ambient ozone by the year 2100 under RCP8.5 scenarios [7]. In this study, we used the NMMAPS dataset of the USA to assess the seasonal variations of ozone effects on mortality in southern and northern communities. Our results indicate that ambient ozone had more pronounced effects on mortality in the autumn season compared to the warm season (spring and summer) in southern communities where average daily temperature is warmer, but the ozone effects were stronger in the warm season than in the cold season (autumn and winter) in northern communities where average daily temperature is cooler. These results are consistent with some previous study findings [20,21,22,23,24]. For instance, Wong et al. observed a 4.0% (95% CI: 1.0% ~ 6.0%) increment of mortality when ambient ozone concentration increased from 10th percentile to 90th percentile in the cold season of Hong Kong that is located in southern China, but the increment was −1.0% (95% CI: −3.0% ~ 2.0%) in the warm season [21]. In a recent study, Jhun et al. also used the NMMAPS dataset to examine the geographic variations of ozone effects in the warm season of 97 US cities [38]. They observed slightly higher effects of ozone in the warm season of the northeastern region than in the southeastern region. However, this study did not investigate the seasonal heterogeneity of ozone effects between different regions [38]. One of our previous studies also demonstrated that ambient ozone had a higher effect on total mortality in the cold season than in the warm season [24].

The mechanisms for different seasonal patterns of ozone effects between the southern and northern communities remain unclear. One hypothesis proposed in some previous studies might provide us some clues, i.e., exposure pattern [13,20,21,22,23,24]. In the southern communities that experience a humid, subtropical climate, particularly the summer season is characterized by hot temperatures and more rain. For example, in the summers of Houston the average maximum daytime temperature was 34 °C, and the average summer rainfall during 1984 to 1999 was 264 mm [39]. Rain can reduce ozone concentration as it reduces sunlight and hence the production of ambient ozone. Moreover, people typically spent time indoors and use air conditioners more frequently to alleviate high temperatures and humidity induced by rain, hence reducing their exposure to ambient ozone. Bell et al. assessed the modification of central air conditioning use on the effects of ozone on mortality in 98 USA communities, and observed that higher prevalence of air conditioning use lessened the effect estimates of ozone [40]. In addition, frequent extreme weather conditions during the summer season (e.g., heavy rains, thunderstorms) were factors that may weaken the relationship between air pollution and mortality [24]. In contrast, the cold season, especially the autumn of southern communities, were milder and had few extreme weather events. For instance, the average TM in autumn in Houston was 21.2 °C. People were therefore more likely to go outdoors and open windows, which might increase their exposure to ambient ozone [21], even though the actual ozone levels during this time were lower (e.g., 41.9 ppb in Houston) than in other seasons (e.g., 44.1 ppb in Houston in summer).

In the northern communities, people are more likely to have different exposure patterns to ambient ozone compared with people in the southern communities due to the climatic differences. In the cold season, people may spend more time indoors due to the bitter cold outdoors and hence reduce their exposure to ambient ozone. For example, in the winter of Syracuse, the average TM was −3.0 °C. However, in the summer season the average TM was 20.8 °C, a temperature more conducive to spending more time outdoors, and hence potentially increasing population exposure to ambient ozone [13,20,21,22,23,24]. In addition, central air conditioning use could also lessen the health effects of ozone [38]. Ambient ozone concentration was also higher in the warm season than in the cold season, which might also strengthen the ozone-related effects on mortality.

Our meta-regression analyses also confirmed the validity of the above findings. We observed that in warm seasons the ozone effects had positive relationships with latitude and negative relationships with average TM across communities, indicating that in the northern communities with milder temperatures people experienced higher mortality risk due to ambient ozone exposure. By contrast, in the winter season, ozone effects had negative relationships with latitude and positive relationships with TM. Our findings are consistent with previous studies [11,41]. It is well known that latitude and temperature are proxies for some other factors, such as outdoor activity, using air-conditioning, change of residence and transportation [41,42], which may affect ozone-related health effects. These results confirm that the variation of the ozone-mortality associations over cities is not random. Understanding the reasons of this spatial variability is important to interpret the “national” ozone effect estimate. In addition, geographic variations of ozone effects on health should be taken into account in future studies, particularly in multi-city studies, otherwise the ozone effects might be either underestimated or overestimated if employing a single overall estimate to make decisions on air quality management.

Choosing the *df* of smoothness of time is an important methodological issue in time-series studies [43]. In most previous NMMAPS studies, the *df* of time was set to 7 per year [32,44,45]. In order to keep the comparability with previous studies, we also used 7 *df* per year for the overall smooth function of time. We further did sensitivity analyses by changing the *df* from 5 to 9 to test the robustness of our results. We observed that our results were generally robust to changing the *df* of time, but the combined ozone-related effects in the southern communities significantly increased while changing the *df* of time from 7 to 9 per year in the winter season. The reasons for this inconsistent finding that existed particularly in the winter season remain unknown, but it may indicate that the ambient ozone effects in the winter of southern communities might have been underestimated in the present study. Some previous studies that were conducted in southern China have also demonstrated that ozone had more pronounced effects on mortality in the cold season than in the warm season [20,21,28,31,42]. On the other hand, the impact of changing *df* of time indicates to us that there may be different patterns of long-term or seasonal trend in the data between seasons. Season-specific *df* of time is necessary to be tested and used in future time-series studies. However, to our knowledge, few studies observed such impacts of *df* of time on the ozone effects between seasons; therefore, more studies are needed in the future to test these results.

There are several limitations of this study. First, the air pollution indices were means from data collected from various monitoring stations. The variance of measurements may differ from station to station, which may lead to measurement error and greater heterogeneity of the results. Second, we observed significant modification effects of latitude on the ozone-mortality associations in the spring and summer seasons even after adjustment for temperature and relative humidity, which indicates that there are still-unexplained heterogeneities between cities. It has been debated that the between-city ozone effect modifiers can be classified broadly into three types: demographic variables (e.g., racial or socioeconomic), variables associated with exposure (e.g., people’s air conditioning use and outdoor activity) and with possible co-pollutants (e.g., SO_2_) [38,41]. However, due to the lack of data availability, many of these between-city effect modifiers were not adjusted for in the meta-regression analyses of latitude. Therefore, more studies are needed in the future to understand the reasons for this intercity variance, which could provide significant information to summarize a “national” ozone effect estimate in cases where there is substantial intercity heterogeneity among the ozone-mortality effects. Thirdly, we did not adjust for other air pollutants such as PM_2.5_, PM_10_, NO_2_ and SO_2_ in the statistical models, which may induce some uncertainties into our results although some previous studies using the NMMAPS data have shown that the ozone effect estimates were not confounded by PM_10_, etc. [11,27]. In addition, infectious disease epidemics in the winter may provide another explanation for the observed seasonal patterns of ozone effects, such as influenza and epidemic cerebrospinal meningitis [46]. However, due to the availability of data, we did not explore the impacts of these infectious disease epidemics on the ozone-related effects. Last but not least, we did not have information on ozone concentrations of indoor environments where people spend most of their time, which may bias the accuracy of effect assessment between ozone exposure and mortality [47].

## 5. Conclusions

The present study revealed different seasonal patterns of ambient ozone effects on mortality between southern and northern communities of the USA. Ambient ozone had more strengthened effects on mortality in the autumn season of southern communities, but had more pronounced effects in the warm season of northern communities. Latitude and average temperature appear to be important modifiers of ambient ozone-related mortality risks. These findings extend our understanding of the short-term effects of ozone on population health, and provide significant reference information for making policy on ambient ozone control and adaptation strategies for protecting people’s health.

## Figures and Tables

**Table 1:** General characteristics of the 20 selected communities in the USA.

	Latitude (°)	Longitude (°)	Population (×100,000)	Average Daily Mortality	Annual Average Temperature (°C)	Annual Average Relative Humidity (%)	Average O_3_ Concentration (ppb)				
							Full Year	Spring	Summer	Autumn	Winter
Southern communities											
Houston	29.8	95.4	34.0	42.8	20.8	71.3	38.9	44.0	44.1	42.0	25.5
Dallas	32.8	96.8	42.0	54.5	19.2	59.8	41.4	45.2	53.7	41.2	25.3
Tampa	28.0	82.5	10.0	18.5	23.0	73.3	41.0	50.1	41.1	39.4	33.4
Shreveport	32.5	93.8	3.5	8.6	18.8	68.9	44.6	48.6	53.3	44.7	31.5
Little Rock	34.7	92.4	3.6	7.9	17.2	67.9	41.1	45.1	53.4	38.8	26.8
St. Petersburg	27.8	82.6	9.2	31.2	23.0	73.3	39.6	49.1	38.5	37.4	33.2
Nashville	36.2	86.8	5.7	11.6	15.6	66.3	34.7	38.9	50.4	31.2	17.8
Austin	30.3	97.8	8.1	8.6	20.8	62.9	40.2	45.4	43.8	42.3	29.2
Jacksonville	30.3	81.7	7.8	14.4	20.4	75.5	41.0	49.7	44.2	38.6	31.4
New Orleans	30.1	89.9	4.8	12.2	20.7	74.8	33.9	41.5	37.2	33.2	23.5
Average	31.3	90.0	12.9	21.0	20.0	69.4	39.6	45.8	46.0	38.9	27.8
Northern communities											
Jersey City	40.7	74.1	6.1	11.5	13.2	62.4	34.3	37.2	56.7	27.4	15.5
New York	40.7	73.9	89.3	190.2	12.8	64.8	32.0	34.1	49.9	24.9	18.8
Syracuse	43.0	76.1	4.5	11.1	9.1	69.8	35.3	41.3	48.0	28.2	23.3
Boston	42.3	71.0	6.9	13.2	10.9	66.5	30.2	35.5	44.3	22.6	18.2
Chicago	41.8	87.7	53.8	115.4	10.1	69.6	28.9	33.0	45.0	22.7	14.4
Newark	40.7	74.2	7.9	18.5	13.2	62.4	28.4	30.7	45.8	20.3	14.6
Pittsburgh	40.4	80.0	12.8	38.3	11.0	65.7	35.8	40.3	55.6	27.0	18.7
Buffalo	42.9	78.9	9.5	25.5	9.2	69.9	35.4	40.0	50.8	27.6	22.7
Washington	38.9	77.0	5.7	15.7	12.8	67.4	32.6	33.7	55.7	25.8	14.7
Rochester	43.2	77.6	7.4	15.8	9.1	71.0	34.1	39.2	48.3	27.1	21.9
Average	41.5	77.1	20.4	45.5	11.1	67.0	32.7	36.5	50.0	25.4	17.9

**Table 2:** Mean and specific percentiles for variables of the 20 study communities in the USA.

	Southern Communities					Northern Communities				
	Mean	Min	25th	75th	Max	Mean	Min	25th	75th	Max
***Full year***										
Total mortality	21.0	7.9	8.6	34.1	54.5	45.5	11.1	12.8	57.6	190.2
Mean temperature (°C)	20.0	15.6	18.4	21.4	23.0	11.1	9.1	9.2	12.9	13.2
Relative humidity (%)	69.2	59.8	65.5	73.7	75.5	67.0	62.4	64.2	69.8	71.0
Maximum 8 h O_3_ (ppb)	39.7	34.5	37.9	41.2	44.7	32.1	28.9	29.8	34.5	35.7
***Spring***										
Total mortality	21.0	8.1	9.3	28.6	54.1	45.4	11.1	13.9	35.5	188.3
Mean temperature (°C)	19.6	15.2	18.6	20.6	22.5	9.9	7.7	8.2	11.6	12.0
Relative humidity (%)	66.8	60.1	64.4	69.0	72.5	63.3	59.4	60.9	65.2	66.8
Maximum 8 h O_3_ (ppb)	45.8	38.9	44.2	49.0	50.1	36.5	30.7	33.8	39.8	41.3
***Summer***										
Total mortality	19.8	7.4	8.3	31.7	50.9	42.5	10.5	12.0	53.3	177.4
Mean temperature (°C)	27.9	25.8	27.4	28.5	29.0	22.3	20.6	20.8	23.9	24.4
Relative humidity (%)	70.1	56.8	66.2	75.9	77.5	67.3	63.8	64.7	69.0	70.7
Maximum 8 h O_3_ (ppb)	46.1	38.1	40.5	53.4	53.7	49.9	44.6	45.2	55.1	56.7
***Autumn***										
Total mortality	20.3	7.7	9.1	26.6	52.4	44.3	10.9	13.4	33.9	184.3
Mean temperature (°C)	20.5	15.9	19.3	21.4	24.0	12.3	10.3	10.7	14.0	14.3
Relative humidity (%)	70.3	59.3	67.2	74.8	80.3	69.0	64.7	66.3	71.0	74.2
Maximum 8 h O_3_ (ppb)	38.9	31.3	37.7	41.8	44.7	25.4	20.3	23.3	27.3	28.2
***Winter***										
Total mortality	23.2	8.7	9.5	37.5	60.8	50.0	12.0	14.0	63.0	211.1
Mean temperature (°C)	11.7	5.2	8.7	14.2	17.2	-0.2	-3.0	-2.5	2.0	2.2
Relative humidity (%)	70.3	62.9	67.2	74.2	75.1	68.2	61.8	61.9	74.6	75.9
Maximum 8 h O_3_ (ppb)	27.8	17.8	25.0	31.9	33.4	18.6	14.3	15.2	21.9	23.1

## Supplementary Materials

The following are available online at www.mdpi.com/1660-4601/13/9/851/s1, Figure S1: Monthly average maximum 8 h O_3_ concentration (ppb) in the 20 study communities in the USA, Table S1: Sensitivity analyses on the impacts (ER, 95% CI) of lag structure on the effects of ozone effects on mortality, Table S2: Changes (%, 95% CI) in ozone-related mortality effect estimates per interval increase in a city-specific characteristic, Table S3: The ERs in mortality for each 10 ppb increment in daily 8 h maximum ozone concentration (lag0-2 day average) in the community with the maximum concentration of ozone in each season.
