# Supplementary material for: Tempo-Spatial Variations of Ambient Ozone-Mortality Associations in the USA: Results from the NMMAPS Data
Source: Int J Environ Res Public Health. 2016 Aug 26;13(9):851. doi: 10.3390/ijerph13090851 (PMC5036684; doi:10.3390/ijerph13090851)
Supplement: Supplementary file 1 [file ijerph-13-00851-s001.pdf]

Supplementary Materials: Temporal-Spatial Variations of Ambient Ozone-Mortality Associations in the USA: Results from the NMMAPS Data

Tao Liu, Weilin Zeng, Hualiang Lin, Shannon Rutherford, Jianpeng Xiao, Xing Li, Zhihao Li, Zhengmin Qian, Baixiang Feng and Wenjun Ma

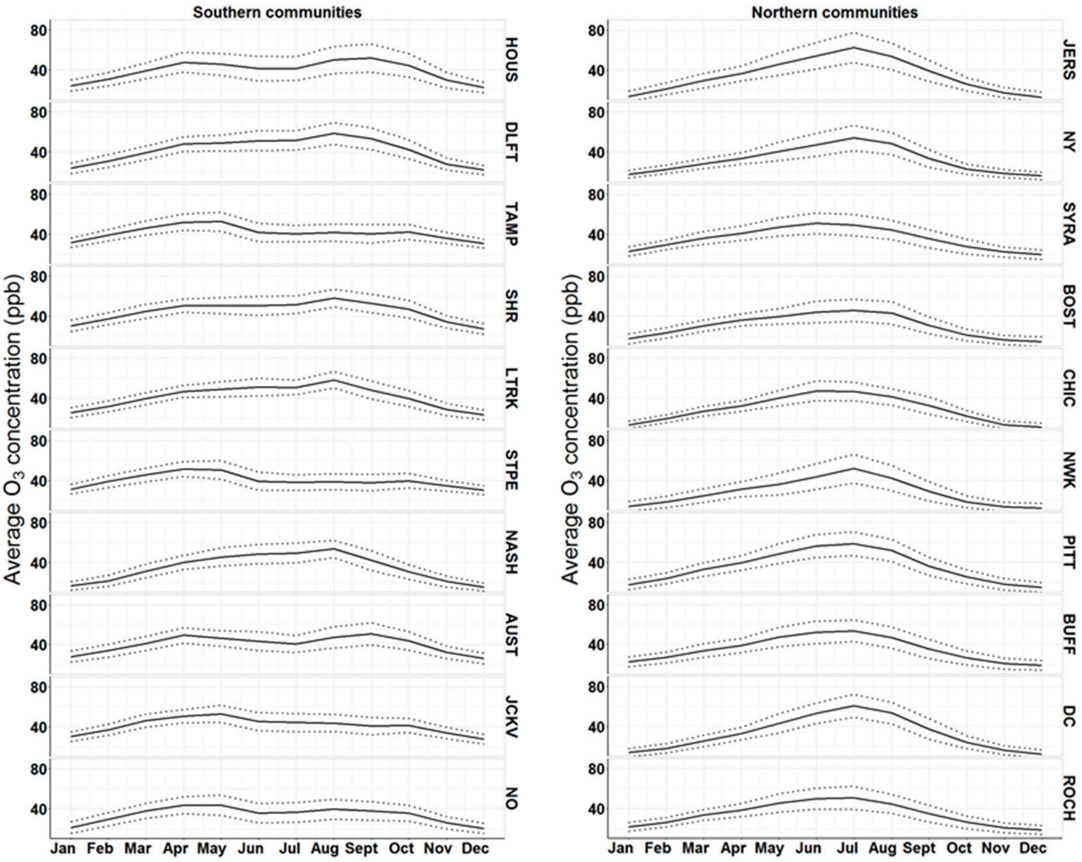

Figure S1. Monthly average maximum 8 h O_3 concentration (ppb) in the 20 study communities in the USA. HOUS: Houston; DLFT: Dallas; TAMP: Tampa; SHR: Shreveport; LTRK: Little Rock; STPE: St. Petersburg; NASH: Nashville; AUST: Austin; JCKV: Jacksonville; NO: New Orleans; JERS: Jersey City; NY: New York; SYRA: Syracuse; BOST: Boston; CHIC: Chicago; NWK: Newark; PITT: Pittsburgh; BUFF: Buffalo; DC: Washington; ROCH: Rochester.

Table S1. Sensitivity analyses on the impacts (Excess risk, 95% CI) of lag structure on the effects of ozone on mortality.

	**Average TM (°C)**	**Southern Communities**			**Average TM (°C)**	**Northern Communities**		
		**Model I**	**Model II**	**Model III**		**Model I**	**Model II**	**Model III**
Spring	19.6	−0.23 (−0.61, 0.12)	−0.07 (−0.49, 0.36)	0.06 (−0.40, 0.53)	9.9	0.68 * (0.33, 1.04)	0.74 * (0.33, 1.16)	0.65 * (0.21, 1.10)
Summer	27.9	−0.08 (−0.37, 0.20)	−0.17 (−0.47, 0.13)	−0.04 (−0.36, 0.29)	22.4	1.14 * (0.92, 1.37)	1.21 * (0.72, 1.71)	1.09 * (0.76, 1.41)
Autumn	20.5	0.36 * (0.04, 0.69)	0.40 * (0.05, 0.75)	0.38 * (0.01, 0.76)	12.3	0.67 * (0.32, 1.03)	0.52 * (0.08, 0.96)	0.27 (−0.21, 0.76)
Winter	11.7	0.30 (−0.22, 0.83)	0.27 (−0.30, 0.84)	0.14 (−0.46, 0.74)	−0.19	−0.52 (−1.10, 0.07)	−0.65 (−1.57, 0.28)	−0.32 (−1.03, −0.40)

Model I: The ERs of lag0-1 day average ozone concentration on mortality after adjustment for daily temperature, time, day of week and relative humidity (RH); Model II: The ERs of lag0-2 day average ozone concentration on mortality after adjustment for daily temperature, time, day of week and RH; Model III: The ERs of lag0-3 day average ozone concentration on mortality after adjustment for daily temperature, time, day of week and RH. *$p < 0.05$.

Table S2. Changes in ozone related morality effect estimates (%, 95% CI) per interval increase in a city-specific characteristic.

	Change (%, 95% CI)
**Latitude (*per 1°*)**	
Spring	9.4 * (4.1, 14.0)
Summer	13.9 * (8.3, 19.7)
Autumn	3.0 (−4.9, 11.6)
Winter	−3.9 (−13.9, 7.3)
**Average temperature (*per 1 °C*)**	
Spring	−8.6 * (−13.9, −3.0)
Summer	−21.3 * (−29.5, −13.1)
Autumn	−3.9 (−12.2, 6.2)
Winter	2.0 (−6.8, 12.7)
**Relative humidity (*per 1%*)**	
Spring	−11.3 * (−18.1, −4.9)
Summer	−6.8 (−13.9, 0.02)
Autumn	−6.8 (−13.9, 1.0)
Winter	−1.0 (−13.1, 11.6)

* $p < 0.05$.

Table S3. The ERs in mortality for each 10 ppb increment in daily 8-h maximum ozone concentration (lag0-2 day average) in the community with the maximum concentration of ozone in each season.

	Southern Communities		Northern Communities	
	Community	ER # (%, 95% CI)	Community	ER # (%, 95% CI)
Spring	Tampa	−0.18 (−1.58, 1.23)	Syracuse	0.97 (−1.25, 3.24)
Summer	Little Rock	−1.58 (−3.47, 0.34)	Washington	1.30 (0.01, 2.60)
Autumn	Dallas	0.82 (0.07, 1.57)	Syracuse	2.11 (−0.34, 4.63)
Winter	Tampa	−0.98 (−2.88, 0.96)	Syracuse	1.23 (−1.45, 3.99)

#: Adjustment for daily TM, time, day of week and RH.
